# Allocentric Versus Egocentric Spatial Memory in Adults with Autism Spectrum Disorder

**DOI:** 10.1007/s10803-018-3465-5

**Published:** 2018-01-29

**Authors:** Melanie Ring, Sebastian B. Gaigg, Mareike Altgassen, Peter Barr, Dermot M. Bowler

**Affiliations:** 1 Autism Research Group, Department of Psychology, City, University of London, Rhind Building, Northampton Square, London, EC1V 0HB UK; 2 Present Address: Department of Child and Adolescent Psychiatry, Medical Faculty of the Technical University Dresden, Dresden, Germany; 3 Donders Institute for Brain, Cognition and Behaviour, Radboud University Nijmegen, Nijmegen, The Netherlands; 4 Department of Psychology, Technische Universität Dresden, Dresden, Germany; 5 Department of Psychology, City, University of London, London, UK

**Keywords:** Spatial navigation, Autism spectrum disorder, Allocentric, Egocentric, Visual short-term memory, Mental rotation, Task support hypothesis

## Abstract

Individuals with autism spectrum disorder (ASD) present difficulties in forming relations among items and context. This capacity for relational binding is also involved in spatial navigation and research on this topic in ASD is scarce and inconclusive. Using a computerised version of the Morris Water Maze task, ASD participants showed particular difficulties in performing viewpoint independent (allocentric) navigation, leaving viewpoint dependent navigation (egocentric) intact. Further analyses showed that navigation deficits were not related to poor visual short-term memory or mental rotation in the ASD group. The results further confirm the need of autistic individuals for support at retrieval and have important implications for the design of signposts and maps.

**Electronic supplementary material:**

The online version of this article (10.1007/s10803-018-3465-5) contains supplementary material, which is available to authorized users.

## Introduction

Autism spectrum disorder (ASD) is a developmental disorder that is characterised by difficulties in the areas of social interaction and communication and restricted and repetitive behaviours (American Psychiatric Association 2013). Individuals with ASD show a heterogeneous cognitive profile with a specific pattern of intact and compromised processes in memory (Boucher and Bowler 2008; Boucher et al. 2012). Rote memory, which is the ability to learn material without understanding its meaning, was found to be a strength of individuals with ASD (e.g. Hermelin and O`Connor 1975). Spared performance was also reported in tests measuring priming (Bowler et al. 1997), immediate cued recall (e.g. Mottron et al. 2001) and recognition memory (e.g. Farrant et al. 1998). Given that these procedures provide more support at test, Bowler et al. (1997) proposed the ‘task support hypothesis’ stating that ASD individuals show less difficulties when they can rely on external sources of support (e.g. recognition rather than free recall of items; Bowler et al. 2004, 2015; Gaigg et al. 2008; Minshew et al. 1992; Mottron et al. 2001; Toichi and Kamio 2003). Bowler et al. (2011) in their *relational binding account* have argued that the reason for this difficulty on unsupported test procedures is a reduced capacity for relational binding in ASD, i.e. difficulties linking elements of experience to one another or to their spatial or temporal context to form a coherent episodic representation in order to enable flexible retrieval of that information. These relational binding difficulties become apparent when ASD participants are asked to remember the context of an item presentation, for example temporal (e.g. Bennetto et al. 1996; Bigham et al. 2010; Gaigg et al. 2014; Minshew and Goldstein 1993; Ring et al. 2016), spatial (e.g. Bowler et al. 2014, 2004; Cooper et al. 2015; Ring et al. 2015, 2016; Semino et al. 2017) or other types of context information (e.g. Hala et al. 2005; Lopez and Leekam 2003; Maister et al. 2013; O’Shea et al. 2005).

Another capacity that is reliant on relational binding is spatial navigation. One way for spatial navigation to be successful is that one needs to create an abstract map representation of the environment which depicts the relation among goal location, object cues and travel direction in the environment. Neurologically, relational binding has been demonstrated as a capacity of the hippocampus (Eichenbaum 2004; Opitz 2010) and also spatial navigation has been shown to be at least in part dependent on the hippocampus (e.g. Bohbot et al. 2004). In particular, allocentric navigation or “shifted view-point representation” refers to navigation dependent on the processing of the relations among goal and landmarks independent of a single view-point and is regulated through the (right) hippocampus (Bohbot et al. 2004). Egocentric navigation or “same view-point representation” on the other hand describes navigation using the self as a reference for navigating a route and is regulated through the caudate nucleus (e.g. Bohbot et al. 2004; Hartley et al. 2004). Following the relational binding account, one would expect specific difficulties with allocentric navigation in ASD, yet few studies have examined this issue.

Investigating processes like memory and spatial navigation in ASD is important since they can give hints to the aetiology of the disorder through comparison with patient populations with other disorders such as amnesia (Feigenbaum and Morris 2004). In addition, difficulties in memory and spatial navigation impact on an individuals’ way to cope with the demands of daily life and ultimately affect their quality of life, which has been reported to be reduced in ASD (e.g. Gilotty et al. 2002; Liss et al. 2001; Van Heijst and Geurts 2015). Previous studies of spatial navigation in ASD show inconsistent results, with two studies reporting no differences between groups (Edgin and Pennington 2005; Caron et al. 2004), one study finding an overall navigation difficulty in ASD (Lind et al. 2013), and two studies finding specific difficulties in allocentric conditions (Prior and Hoffmann 1990; Lind et al. 2014), which is what would be predicted following the relational binding account (Bowler et al. 2011). It is important to note that only two of these earlier studies compared allocentric and egocentric conditions within one study (Lind et al. 2013, 2014). The paradigm used by Lind et al. (2013, 2014) presented participants with target objects on a virtual island that participants were asked to navigate to. In an egocentric/visible condition, locations of target objects were marked by flags, whereas in an allocentric condition, participants had to relocate the objects without the flags. For this task, one could argue that presenting participants with flags as cues to the hidden objects can be seen as task support and since people with ASD typically perform better on supported tasks (e.g. Bowler et al. 1997, 2004), this may have been the reason for their better performance on the visible trials. The present study was designed to address this gap in the existing literature by equating egocentric and allocentric conditions on their relational binding requirements.

In order to systematically compare adults with and without ASD on allocentric and egocentric spatial navigation, we adapted a computerized version of the Morris Water Maze, in which participants were asked to find a hidden platform in a virtual swimming pool, which was surrounded by object cues (Feigenbaum and Morris 2004). One of the previous spatial navigation studies in ASD referred to above also used a water maze paradigm (Edgin and Pennington 2005). However, the results of that study are inconclusive because the authors tested participants only with a place learning condition, i.e., testing simple spatial memory that enabled the use of allocentric as well as egocentric processing (Burgess 2006). As a consequence, ASD participants may have compensated for potential allocentric problems by using intact egocentric processing. To overcome this issue, the improved design by Feigenbaum and Morris (2004) was employed in the current study, which is described in detail in what follows. After a familiarisation phase called place learning, where object cues, platform location and the participant stayed in the same position across a number of trials, allocentric and egocentric conditions were presented in which either object cues or the participant changed their position. In the allocentric condition, the object cues and the platform position were fixed, however, the participant moved around the screen keeping the relations among platform location and cues constant. In the egocentric condition, the platform and the participant stayed in the same position but the object cues changed position to disturb allocentric processing. Feigenbaum and Morris tested 14 patients with a right unilateral temporal lobectomy (RTL), 16 patients with a left sided one (LTL), and 16 healthy control participants on this task and found that only the RTL group showed impaired performance in the allocentric condition. In the current study, two additional conditions (allocentric and egocentric 2) were added to control for the possibility that group differences in the Feigenbaum and Morris study resulted from differences in task features between the allocentric and egocentric conditions whereby in one condition the person moved (allocentric 1) and in the other condition the platform moved (egocentric 1). In the two added conditions, either the participant moved together with the platform position (allocentric 2) or the platform moved with the objects and the participant stayed in the same position (egocentric 2). In addition, we extended the number of trials on each task from 8 to 16 to enable better learning of the platform position. This takes into consideration that ASD individuals might need more repetitions to reach the same performance level as TD individuals (see Bowler et al. 2008). Finally, we implemented place learning as the first condition, followed by the allocentric and egocentric conditions in counterbalanced order across participants. Feigenbaum and Morris (2004) always asked participants to perform place learning followed by the allocentric condition, which was then followed by another place learning and the egocentric condition, which may have resulted in unwanted order effects.

Similarly to Feigenbaum and Morris (2004), we expected that participants in both groups would show learning across trials for all conditions and that adults with ASD would show particular difficulties in allocentric navigation, leaving egocentric navigation intact. Further, we expected a similar pattern of results for the two added conditions with individuals with ASD showing difficulties in allocentric 2 but not egocentric 2 compared to the TD group. In addition to the Morris Water Maze task, three tasks to assess participants’ visual short-term memory and mental rotation were administered (Feigenbaum and Morris 2004). This was to explore whether people with ASD show difficulties with the temporary storage and manipulation of spatial information per se, which would point to additional difficulties related to functions based outside the hippocampus, for example involving parietal brain regions (Silk et al. 2006; Tadi et al. 2009; Zacks 2008). Following the relational binding account (Bowler et al. 2011), we did not expect differences between groups on tests of visual short-term memory and mental rotation.

## Methods

### Participants

Sample size was determined by reference to previous studies of this kind and via power calculation. Feigenbaum and Morris (2004) included 14–16 participants in each of their three groups of participants. In addition, power calculation using G*Power (Faul et al. 2007) showed that 16 participants in each group would be needed to find the predicted between-group difference in allocentric navigation with an effect size of η_p_^2^ = .10 and a power of .95. To increase statistical power because of the heterogeneity of ASD samples, 26 individuals with ASD (23 men, M_age_ = 38.81 years, age range 24–63 years) and 26 TD individuals (18 men, M_age_ = 42.12 years, age range 22–61 years) took part. Groups were matched on gender, X^2^ = 2.88, *p* = .09, chronological age (CA), Verbal Intelligence Quotient (VIQ), Performance IQ (PIQ) and Full-scale IQ (FIQ) as measured by the third edition of the Wechsler Adult Intelligence Scale (WAIS-III^UK^; The Psychological Corporation 2000; see Table 1). Participants were randomly selected from a panel of over 50 individuals with whom the Autism Research Group is in regular contact. Initially, the panel was created through advertisements in newspapers, job agencies, contacts to self-help groups for individuals with ASD and word of mouth. In addition, TD participants were recruited through newspaper advertisements. ASD individuals were all diagnosed by experienced clinicians according to DSM-IV-TR criteria (American Psychiatric Association 2000) prior to the study. In addition, 23 of these individuals were available to take part in an assessment with the Autism Diagnostic Observation Schedule (ADOS; Lord et al. 1989) done by researchers trained to research reliability standards on this instrument (for ADOS scores, see Table 1). TD individuals were only included in the study if they were free of psychotropic medication and had no personal or family history of neuropathology or psychiatric illnesses. All participants were born in the UK and spoke English as their mother tongue. Informed consent was obtained from all individuals before taking part, they were paid standard university rates for their participation and their travel costs were paid for. The experimental procedures outlined below adhere to the ethical guidelines set out by the British Psychological Society and were approved by City, University of London’s Research Ethics Committee.

**Table 1:** Descriptive statistics for individuals with autism spectrum disorder (ASD) and typically developing (TD) individuals

Measure	ASD (23m, 3f)		TD (18m, 8 f)		*t*(50)	*p*	Cohen’s *d*
	*M*	*SD*	*M*	*SD*			
Age (years)	38.81	11.82	42.12	12.14	1.00	.32	.28
VIQ^a^	109	16.6	111	16.3	.54	.59	.15
PIQ^b^	108	19.6	107	17.6	.22	.83	.06
FIQ^c^	110	18.7	110	17.9	.18	.86	.05
ADOS-C^d^	2.74 (0–5)	1.39					
ADOS-RSI^e^	6.74 (3–13)	2.96					
ADOS-Total^f^	9.48 (3–17)	3.72					
ADOS-I^g^	1.19 (0–2)	.60					
ADOS-SB^h^	1.41 (0–5)	1.30					

^a^Verbal IQ (WAIS-III^UK^)

^b^Performance IQ (WAIS-III^UK^)

^c^Full-scale IQ (WAIS-III^UK^)

^d^ADOS—communication subscale

^e^ADOS—Reciprocal social interaction subscale

^f^ADOS total score − communication + reciprocal social interaction

^g^ADOS—imagination/creativity subscale

^h^ADOS—stereotyped behaviours and restricted interests. ADOS scores are presented with range in brackets

### Materials and Procedure

Participants were tested individually and testing took about 1.5 h. The order of tasks was counterbalanced across participants, with the ASD and TD members of each matched pair receiving the same order. Visual short-term memory and mental rotation tests were either given before or after the Water Maze task, which was counterbalanced across participants.

#### Spatial Navigation Task

**Fig. 1:**
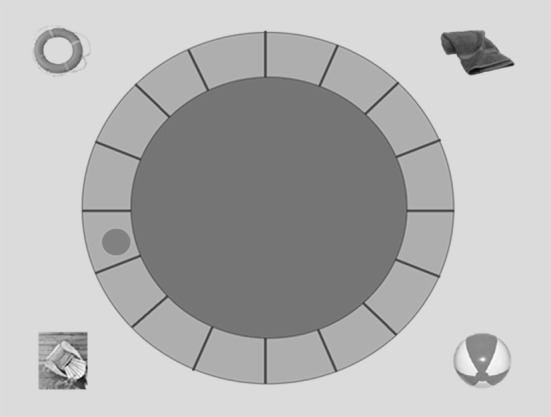
Example of the screen used

A computerized version of the Morris Water Maze written in Microsoft Visual Basic 6 was used to measure spatial navigation. This was an adaptation of Feigenbaum and Morris’ task (2004). The task was presented on a 19″ touch-sensitive screen (http://www.elotouch.com/Products/LCDs/1939L/), which was placed horizontally on a table located in a soundproof room. The table was surrounded by a small area, which was separated from the rest of the room by beige curtains placed on the ceiling and hanging from the ceiling forming a little cubicle to reduce the influence of external distracters or cues such as windows or features on the walls within the room to guide navigation. During the task, participants were asked to stand around the table looking down on the screen and to place their finger on it. There was sufficient space in the cubicle to allow participants to walk around the horizontal screen according to the task instructions. During task performance, room lights were turned off so that the only visible light came from the screen. This further reduced the influence of features in the room. On each trial, participants were presented with a virtual swimming pool environment. The display included a blue circle area representing the water in the pool, which was surrounded by an orange wall representing the wall around the pool. Outside the pool, a green area was presented representing grass growing around the pool area. On the grass, four objects were displayed in each corner of the screen, namely a chair, a life ring, a towel and a beach ball (see Fig. 1). The starting point, the location where participants were supposed to put their finger before moving it in the pool area, was indicated by a red dot on the orange wall around the swimming pool. On every trial, participants were asked to move their finger across the blue pool area until the platform appeared, which was represented by a brown box.

Participants were told that their task was to work out and learn the shortest way towards a hidden platform in the pool over several trials starting at the starting point and moving their finger across the blue space without lifting it from the screen and without crossing the orange perimeter line until the hidden platform appeared. The position of the participant’s finger was recorded during the entire experiment. After three practice trials during which participants learned how to use the touch-sensitive screen, participants were first presented with the place learning block and then with the 2 *allocentric* and the 2 *egocentric* blocks. Blocks were presented in counterbalanced order across participants with two individuals of a matched pair (one ASD and one TD person with similar IQ) performing the same order.

The starting point changed position on the orange wall in a random order. Each block consisted of 16 trials each lasting 60 s. If a participant could not find the platform within 60 s in one trial, a time out message together with the platform appeared on the screen and the participant was asked to move their finger to the platform. Each trial was followed by a distracter task, which consisted of a series of 12 blue circles (‘bubbles’) that appeared one after the other at random locations on a black screen and the participant was asked to ‘burst’ the bubbles by touching them. Measures taken were path length, time to find the target, path angle and percentage of time in the target quadrant. The target quadrant was defined as the quadrant where the participants’ path entered the platform. Control measures taken were: out of pool times (number of times participants left the pool area), finger lift times (number of times participants lifted the finger from the screen), time out times (number of times participants did not reach the target within 60 s), time before first movement (time from touching the starting point until the first movement in the pool area), and the duration of the distracter task (time for distracter task).

*Place learning* served as a control condition without any systematic manipulations taking place. The platform, the object cues and the participant stayed in the same location across the 16 trials of the task.

In the *allocentric 1* condition (original), the platform and the object cues were presented in the same locations across the 16 trials but the participant had to move to another side of the table after every trial in a fixed randomized order. The direction of movement was indicated by an arrow on the screen and the phase ‘Please move to this side’. The movement could be 90 (in both directions) or 180° and it was used to disrupt egocentric processing.

The *egocentric 1* condition (original) was designed to disturb allocentric processing in that the platform and the participant stayed in the same position across the 16 trials, but the object cues rotated in a fixed randomized order after every trial. Again, the movement could be 90° (in both directions) or 180°. The participant, however, did not see the objects rotate; the objects were only presented in their new rotated order to the participant.

In the *allocentric 2* condition, participants stayed in the same position but the platform moved with the objects so that the relations among platform and objects stayed the same. This was to disrupt egocentric processing. The movement was 90° (in both directions) or 180°.

In *egocentric 2*, allocentric processing was disrupted in that the objects stayed in their same positions and did not relate to the platform position. The platform moved with the participants’ position, so that the relation between platform and participants position stayed the same.

#### Visual Short-Term Memory and Mental Rotation

To measure participants’ visual short-term memory, a version of the *Brooks matrix task* (Brook 1967) was used. Participants were presented with sentences describing spatial relations of numbers in a grid. The grid was a 4 × 4 matrix with 16 squares. The sentences were formulated as prompts describing in which cells of the grid which numbers were to be put to encourage mental imagery of the numbers in the grid cells. Participants were asked to repeat the sets of sentences verbatim (e.g. ‘In the starting square put a 1. In the next square up put a 2.’). The task started with sets of two sentences describing the positions of two numbers in the grid. The last sets included eight sentences. Participants were given three trials at every level and the task stopped if they had got three trials wrong at one level or completed all eight levels. Dependent variables were the maximum level achieved (2–8), the number of correct trials (up to 24) and the number of trials attempted (up to 24).

#### Mental Rotation

Participant’s ability for mental rotation was assessed with the *Manikin Task* (Ratcliff 1979; see also: Acker and Acker 1982) and the *Mental Rotations test* (Peters et al. 1995). The *Manikin Task* was adapted with E-Prime software (http://www.SciencePlus.nl/E-Prime) for presentation on a 15″ screen. Participants` task was to indicate if the depicted little man on the screen was holding a black disc in *his* left or in *his* right hand. The man was presented with his front or back to the participant as well as right way up or standing on his head. Measures of performance were reaction time and accuracy. Before and after this task, participants’ were asked to complete a control task to assess their ability to make simple left/right judgements (see Ratcliff 1979; Acker and Acker 1982). In that task, participants were presented with two circles on the screen (one black and one white), which were separated by a horizontal black line and they were asked to indicate as quickly as possible if the black circle was presented on the right or the left side of the horizontal line.

In the *Mental Rotations task*, (version A from Peters et al. 1995, paper and pencil test) participants were presented with 3D objects made from ten blocks, presented from different angles and their task was to pick two out of four figures that matched a target figure. Dependent measures were the sum of credits (1 credit for 2 correct stimulus figures for an item, no credit was given if the participant chose one incorrect figure that did not match the target figure) and the number of trials attempted (out of 24).

## Results

The data were analysed using Chi-Squared test for nominal data, t-tests, repeated measures ANOVAs, point biserial and bivariate correlations. If the Sphericity assumption was violated, Greenhouse-Geisser correction (GG) was applied. In the case of significant differences, Bonferroni-corrected post hoc tests were conducted. The significance level was set at .05 for all tests.

### Spatial Navigation—Morris Water Maze

All data are presented in Table 2. In addition, to show learning over trials for the different conditions, Figure S1 in the supplementary materials presents heat maps showing a comparison of the paths for each group between the first and the last trial of every condition. All analyses presented here focus on the percentage of time spent in the target quadrant as this is seen as the most suitable measure for this kind of analysis. The data on all other measures including control measures for allocentric and egocentric conditions are presented in the supplementary materials and Tables S1–S3.

**Table 2:** Percentage of time in target quadrant for place learning, allocentric 1, egocentric 1 (original conditions), allocentric 2 and egocentric 2 (added conditions) for individuals with autism spectrum disorder (ASD) and typical development (TD)

Measure	Condition	ASD		TD		Total	
		*M* (range)	*SD*	*M* (range)	*SD*	*M* (range)	*SD*
Percentage of time in target quadrant	Place learning	44.28 (0–100)	34.31	48.51 (0–100)	35.51	46.39 (0–100)	34.96
Percentage of time in target quadrant	Allocentric 1 (quadrant 4)	28.87 (0–100)	29.74	34.46 (0–100)	32.17	31.67 (0–100)	31.09
	Egocentric 1 (quadrant 2)	37.57 (1–100)	32.79	36.93 (0–100)	31.32	37.25 (0–100)	32.05
	Total	33.22 (0–100)	31.59	35.70 (0–100)	31.75	34.46 (0–100)	31.68
Percentage of time in target quadrant	Allocentric 2	35.75 (0–100)	28.88	36.09 (0–100)	29.29	35.92 (0–100)	29.07
	Egocentric 2	27.96 (1–100)	26.58	29.86 (0–100)	28.12	28.91 (0–100)	27.36
	Total	31.86 (0–100)	28.01	32.98 (0–100)	28.86	32.42 (0–100)	28.43

### Place Learning

Place learning was used to ensure that participants in both groups were able to use the equipment properly and that they show learning over trials. The data were analysed using a 2 (Group [ASD, TD]) × 16 (Trial [1–16]) repeated measures ANOVA, which showed a significant main effect of *Trial*, F(8.55,427.52) = 40.30, p < .0001, η_p_^2^ = .45, GG. No Group main or Group × Trial interaction effects were significant, *F*_max_ < 1.66, *p*_min_ > .20, η_p_^2^_max_ < .04, confirming similar learning across trials for both groups. Similar results were found when analysing the data for the three other measures (path length, time to target, path angle, see Table S1).

### Allocentric 1 vs. Egocentric 1

The data were analysed using a 2 (Group [ASD, TD]) × 16 (Trial [1–16]) × 2 (Condition [allocentric, egocentric]) repeated measures ANOVA. Next to a significant main effect of *Trial, F*(8.92,446.23) = 88.31, *p* < .0001, η_p_^2^ = .64, GG, as well as a significant *Trial* × *Condition* interaction, *F*(8.05,402.54) = 74.70, *p* < .0001, η_p_^2^ = .60, there was also a significant main effect of *Condition, F*(1,50) = 13.18, *p* < .001, η_p_^2^ = .21, showing that percentage of time spent in the target quadrant increased and was higher for the egocentric compared to the allocentric condition for most trials. A significant *Group* × *Condition* interaction, *F*(1,50) = 4.10, *p* < .05, η_p_^2^ = .08, showed that the ASD group spent a shorter percentage of time in the target quadrant compared to the TD group in the allocentric (*p* < .05, Cohen’s *d* = .59) but not the egocentric condition (*p* = .82, Cohen’s *d* = .06). There was no significant main effect of Group, *F*(1,50) = 1.20, *p* = .28, η_p_^2^ = .02,

### Allocentric 2 vs. Egocentric 2

The data were analysed using a 2 (Group [ASD, TD]) × 16 (Trial [1–16]) × 2 (Condition [allocentric 2, egocentric 2]) repeated measures ANOVA. There was a significant main effect of *Condition, F*(1,50) = 28.54, *p* = .00, η_p_^2^ = .36, with a higher percentage of time spent in the target quadrant for the allocentric 2 compared to the egocentric 2 condition. However, this was the case for both groups as there was no main effect of Group, F(1,50) = .28, p = .60, η_p_^2^ = .01, or Group × Condition interaction, *F*(1,50) = .36, *p* = .55, η_p_^2^ = .01.

Because of the slight difference in the number of men and women in each group who participated in the task and previous reports of TD women performing worse than men at spatial navigation (Astur et al. 2004), we investigated how gender might have affected the results of the current study. Point biserial correlation analyses showed that there were significant positive relations between gender and performance on the allocentric 1 (r = .32, *p* = .02) and the allocentric 2 (r = .37, *p* = .008) conditions indicating that women performed better than men in both allocentric conditions. There were no significant relations between gender and either of the egocentric conditions (r_max_ < .14, *p*_min_ > .34) making it unlikely that the slight difference in gender between groups may have hindered the detection of a between-group difference in performance on the egocentric conditions. In addition, including gender as a covariate in an ANCOVA repeating the analyses reported above showed that the direction of results stayed the same.

### Visual Short-Term Memory and Mental Rotation

The data are presented in Table 3, and they were analysed using independent samples t-tests. There were no significant differences in any of the measures for any of the tasks.

**Table 3:** Measures for visual short-term memory and mental rotation for participants with autism spectrum disorder (ASD) and typical development (TD)

Task	ASD *M *(*SD*)	TD *M *(*SD*	*t*(*df*)	*p*	Cohen’s *d*
Brooks matrix task^a^					
Total correct (out of 24)	12.92 (4.87)	12.04 (5.30)	.61 (48)	.54	.17
Maximum level (out of 8)	6.12 (1.62)	6.08 (1.96)	.08 (48)	.94	.02
Trials attempted (out of 24)	17.52 (3.94)	17.28 (5.00)	.19 (48)	.85	.05
Manikin task^b^					
Average accuracy	.91 (.14)	.83 (.16)	1.89 (46)	.07	.55
Average RT in ms	2964.46 (1215.11)	3195.15 (1144.24)	.68 (46)	.50	.20
Mental rotations test					
Sum of credits (out of 24)	8.08 (6.09)	7.04 (4.51)	.70 (50)	.49	.19
Trials attempted (out of 24)	15.23 (5.70)	14.42 (5.11)	.54 (50)	.59	.15

^a^Only 25 TD and 25 ASD individuals completed this task

^b^One ASD individual did not complete this task. A further 2 ASD and 1 TD participants were excluded because they were at chance in discriminating between right and left in the control task. For both tasks, the remaining participants in both groups were still matched on VIQ, PIQ, FIQ, age (*t*_max_ = -.81, *p*_max_ = .42, Cohen’s *d*_max_ = .23) and gender (*X*^*2*^_max_ = 2.93, *p*_max_ = .09).

### Correlations Among Tasks

Finally, we investigated correlations among allocentric navigation performance and performance on memory and mental rotation tasks. Since there were no significant correlations among any of the measures for either group (see Table 4), it seems unlikely that performance on the visual short-term memory and mental rotations tasks may have influenced performance on the allocentric navigation task.

**Table 4:** Bivariate correlations among spatial navigation performance in the allocentric condition and performance on visual short-term memory (Brooks matrix task) and mental rotation tests (Manikin, Mental rotations task) for groups of individuals with autism spectrum disorder (ASD) and typical development (TD) and for both groups in total

	Allo ASD	Allo TD	Allo Total
Brooks matrix task—total correct	− .10	.33	.13
Brooks matrix task—maximum level	− .22	.21	.06
Brooks matrix task—trials attempted	− .22	.17	.03
Manikin task—average accuracy	.36^+^	.19	.19
Manikin task—average RT	− .25	− .25	− .20
Mental rotations test—sum of credits	.11	.20	.11
Mental rotations test—trials attempted	.26	− .21	− .04

^+^*p* < .10

## Discussion

The primary aim of this study was to explore if ASD individuals show spatial navigation difficulties particularly in allocentric spatial navigation. Such a deficit would be consistent with the relational binding account of autistic memory (Bowler et al. 2011). To test this, we compared matched groups of adults with and without ASD on navigation conditions that either required egocentric or allocentric processing (Bohbot et al. 2004) using a human virtual reality adaptation of the Morris Water Maze task. We predicted to find particular difficulties in ASD with forming view-point independent, allocentric representations. As control tasks visual short-term memory and mental rotation tasks were used to measure participants’ ability to process and manipulate spatial information. We did not expect difficulties in ASD on these tasks.

Our prediction was confirmed for the two original test conditions. Only for the allocentric condition ASD individuals spent less time in the target quadrant compared to TD individuals. This finding is supported by a number of other observations. First, there were no differences between groups in place learning (the baseline condition). Second, there were no differences between groups in participants’ ability to follow instructions (out of pool, finger lift times) and speed of learning (time out times). There was also no difference between groups in how long they took to complete the distracter task between blocks (bursting the bubbles), suggesting that both groups experienced the same time interval between tasks. Finally, consistent with our expectations no between-group differences were found for the control tasks of visual short-term memory and mental rotation. The absence of any significant correlations among allocentric navigation and visual short-term memory and mental rotation performance makes it unlikely that these abilities had any influence on the significant between-group difference in allocentric spatial navigation performance.

There are a number of possible caveats to the conclusions drawn from the present findings. These caveats ask for a cautious interpretation of the results and future research is needed to confirm them and the conclusions drawn from this study. First, the allocentric condition of our task may have been more complex than the egocentric condition which was reflected in participants’ longer paths and time taken to find the platform in the allocentric compared to the egocentric condition. However, the formation of a viewpoint independent representation of the world is in general a more complex operation. What is important, however, is the fact that the allocentric condition was more difficult for both groups, yet only ASD participants showed diminished performance in this condition relative to the comparison group. Second, it might seem surprising that we did not find any differences between groups in any of the other performance measures such as time to target or path length. However, these other measures are not independent of one another and, therefore, they do not necessarily indicate success in the task. A very high variation among participants might have obscured any differences between groups in these performance measures. Percentage of time in the target quadrant is a measure that is often used in the literature and it is less vulnerable to variation between participants because it is expressed as a percentage. A third limitation may be that more females were tested in the TD compared to the ASD group. Since it is known that females show lower performance compared to males on spatial navigation tasks in TD populations (Astur et al. 2004), the slightly higher number of females in our TD sample may have diminished differences between groups. This is however unlikely as correlations between task performance and gender suggest that women performed better on the allocentric conditions in this study. A fourth caveat may be that an aerial view on the search area was used which makes the task less realistic and might require different processing mechanisms compared to navigation in the real world. Future studies should, therefore, use real-life navigation or 3D environments to represent real-life situations more closely. Despite this last limitation, the Water Maze task was chosen to be most appropriate since the aim of this study was to test the relational binding account of ASD which implicates altered functioning of the hippocampus as the basis of difficulties in ASD. Moreover, Feigenbaum and Morris (2004) showed difficulties in allocentric navigation on the water maze task in a patient population with temporal lobectomy including the hippocampus.

At first glance, it may also seem surprising that there was no significant difference between groups in the percentage of time spent in the target quadrant for the added allocentric condition (allocentric 2). In the two added conditions, both groups spent more time in the target quadrant in allocentric 2 compared to egocentric 2. Also, the results suggested that the egocentric 2 condition was more difficult for both groups. In this condition, the platform position moved when the participant moved, which is unnatural and does not happen in real life because buildings stay where they are in space. However, both groups could perform this condition and most importantly the ASD group performed as well as the TD group which indicates that their spatial awareness in relation to the environment is sufficiently robust to perform the task. Allocentric 2 may have put fewer demands on the ASD group than allocentric 1 during which participants had to move around the pool while the platform and object positions remained fixed. When moving around the display area, the participant saw the display from a different perspective on every trial and had to mentally rotate the display back to the original perspective. In contrast, in allocentric 2 participants stayed in their original location while the platform and the objects changed their position and relations between platform and object positions stayed the same. Here, participants did not need to mentally rotate the display because it was being rotated for them. Possibly, allocentric 1 put higher demands on perspective taking abilities than allocentric 2. Indeed in typical participants it has been found that perspective taking abilities predicted spatial navigation abilities in a pointing task (Kozhevnikov et al. 2006). Alternatively, by alleviating the demands for mental rotation, the allocentric 2 condition may have provided more support than allocentric 1 and this additional ‘Task Support’ (Bowler et al. 2004) may be the reason why difficulties in the ASD group were only apparent in the unsupported allocentric 1 condition. Possibly, ASD individuals can overcome their difficulties under certain environmental conditions (see also Gaigg et al. 2008). Differences between allocentric 1 and 2 may also have resulted from differing task complexity, since ASD individuals have been shown to struggle when tasks are more complex (Minshew and Goldstein 1998). Complexity can be operationalised in terms of the number of relations/bindings a participant needs to form in each task. For allocentric 2, binary relations (Halford 1993) between the objects and the platform position need to be formed (there are no changes of objects’ positions independently of each other, therefore, 2 binary relations are enough). For allocentric 1, the participant needs to form a ternary relation between the platform and object positions and their own position in order to locate the platform in a given trial. Possibly, ternary as opposed to binary relations (Halford 1993) are particularly difficult for ASD individuals (Boucher and Bowler 2008).

Overall our findings add further support to recent evidence showing that ASD individuals have difficulty with allocentric spatial navigation as evidenced by spending less time in the target quadrant of a navigation task (Lind et al. 2013). The fact that we could replicate this finding with a different paradigm suggests that this is a robust effect. Given that ASD individuals performed similarly or even slightly better than TD individuals on the visual short-term memory and mental rotations tasks provides strong support for the idea that hippocampally mediated processes rather than more general difficulties with temporal storage and manipulation of spatial information lead to a deficit in the allocentric navigation condition. Therefore, our finding expands the relational binding account in ASD (Bowler et al. 2011) to spatial navigation and further reinforces the task support hypothesis (Bowler et al. 2004) in a spatial navigation paradigm. The present findings have important implications for the design of signposts and maps. Given that individuals with ASD show unimpaired performance in egocentric conditions, they might benefit from map displays along roads and at bus stops that are presented in the direction in which an individual is walking.

## Electronic Supplementary material

Below is the link to the electronic supplementary material.

Supplementary material 1 (DOCX 2353 KB)
