# Supplementary material for: Allocentric Versus Egocentric Spatial Memory in Adults with Autism Spectrum Disorder
Source: J Autism Dev Disord. 2018 Jan 29;48(6):2101–11. doi: 10.1007/s10803-018-3465-5 (PMC5948263; doi:10.1007/s10803-018-3465-5)
Supplement: Supplementary file 1 — Supplementary material 1 (DOCX 2353 KB) [file 10803_2018_3465_MOESM1_ESM.docx]

**Supplementary Materials**

**Place learning.** The data for the length of the path to find the platform (path length), the duration of time needed to find the platform (time to target) and the angle of the path taken heading towards the platform after first movement on the screen (path angle) for the baseline condition are presented in Table S1. To calculate the measure path length, the shortest path length was subtracted from the actual path length in any given trial to enable a comparison between different trials. This measure was then transformed from pixel into mm. Because of a very high variation in the data time to target, path angle and path length were square root transformed. All data were analysed using 2 (Group [ASD, TD]) x 16 (Trial [1-16]) repeated measures ANOVAs. There was a significant main effect of *Trial* for all the measures indicating learning over trials. However, there was no main effect of Group or Trial x Group interaction in any of the measures, showing that both groups learned at similar rates (see Table S1).

[Insert Table S1 here]

**Allocentric 1 vs. egocentric 1.** The data are presented in Table S2. Path length, time to target and path angle were analysed with 2 (Group [ASD, TD]) x 16 (Trial [1-16]) x 2 (Condition [allocentric 1, egocentric 1]) repeated measures ANOVAs. Significant main effects of *Condition* with longer paths and durations to find the target for the allocentric 1 compared to the egocentric 1 condition indicated that this condition was harder and the absence of any main effects of Group or Group x Condition interactions confirmed that this was the case for both groups.

**Allocentric 2 vs. egocentric 2.** The data for the two added conditions are presented in Table S2. Measures were analysed using 2 (Group [ASD, TD]) x 16 (Trial [1-16]) x 2 (Condition [allocentric 2, egocentric 2]) repeated measures ANOVAs. There was a significant main effect of *Condition* for path angle showing that egocentric 2 may have been more difficult than allocentric 2. However, the absence of significant differences between groups or interactions of Group x Condition for any of the measures indicated that if any differences in difficulty level between the conditions existed that they were similar for both groups.

[Insert Table S2 here]

**Control measures.** The data are presented in Table S3. All measures were analysed using 2 (Group [ASD, TD]) x 16 (Trial [1-16]) x 4 (Condition [allocentric 1, egocentric 1, allocentric 2, egocentric 2]) repeated measures ANOVAs. There were no significant differences between groups in the number of times participants moved their fingers outside the blue pool area (out of pool), lifted their finger from the touch-sensitive screen (finger lift), the number of times participants could not find the platform within 60 sec (time out times), or the time from first touching the red dot on the orange wall around the pool until the first movement in the blue pool area (first movement). A significant main effect of *Condition* for the duration of the distracter task (time for distracter task) indicated that participants needed more time to complete the distracter task after allocentric 1 and egocentric 2 conditions.

[Insert Table S3 here]

Table S1

*Descriptive and inferential statistics for additional measures taken in Place learning (baseline condition) for individuals with autism spectrum disorder (ASD) and typical development (TD).*

|  | **Descriptive statistics** | | | | | | **Inferential Statistics** | | | | | | | | |
| --- | --- | --- | --- | --- | --- | --- | --- | --- | --- | --- | --- | --- | --- | --- | --- |
|  | **ASD** | | **TD** | | **Total** | | **Group** | | | **Trial** | | | **Trial x Group** | | |
| **Measure** | ***M***  **(range)** | ***SD*** | ***M***  **(range)** | ***SD*** | ***M***  **(range)** | ***SD*** | ***F*(df)** | ***p*** | **η_p_^2^** | ***F*(df)** | ***p*** | **η_p_^2^** | ***F*(df)** | ***p*** | **η_p_^2^** |
| **Actual-shortest path length in mm (sqrt)** | 13.16  (0-95) | 15.00 | 11.08  (0-76) | 12.92 | 12.12 (0-95) | 14.03 | *F*(1,50) = 0.81 | .37 | .02 | ***F*(7.87,**  **393.63) = 9.21 GG** | **.00** | **.16** | *F*(7.87,  393.63) = 0.72 GG | .68 | .01 |
|  |  |  |  |  |  |  |  |  |  |  |  |  |  |  |  |
| **Time to target in ms (sqrt)** | 61.24 (13-267) | 41.68 | 55.05 (9-253) | 37.37 | 58.14 (9-267) | 39.68 | *F*(1,50) = 1.09 | .30 | .02 | ***F*(7.24, 362.13) = 19.80 GG** | **.00** | **.28** | *F*(7.24, 362.13) = 0.73 GG | .65 | .01 |
|  |  |  |  |  |  |  |  |  |  |  |  |  |  |  |  |
| **Path angle (sqrt of absolute value)** | 5.08  (0-13) | 2.98 | 4.81  (0-13) | 2.93 | 4.95  (0-13) | 2.96 | *F*(1,50) = 0.38 | .54 | .01 | ***F*(10.22,511.07) = 5.34 GG** | **.00** | **.10** | *F*(10.22,511.07) = 1.72 GG | .07 | .03 |

Table S2

*Descriptive and inferential statistics for additional measures taken for allocentric 1, egocentric 1 (the 2 original conditions), allocentric 2 and egocentric 2 (the 2 added conditions) for individuals with autism spectrum disorder (ASD) and typical development (TD).*

|  |  | **Descriptive statistics** | | | | | | **Inferential Statistics** | | | | | | | | |
| --- | --- | --- | --- | --- | --- | --- | --- | --- | --- | --- | --- | --- | --- | --- | --- | --- |
|  |  | **ASD** | | **TD** | | **Total** | | **Group** | | | **Condition** | | | **Group x Condition** | | |
| **Mea-sure** | **Con** | ***M* (range)** | ***SD*** | ***M* (range)** | ***SD*** | ***M* (range)** | ***SD*** | ***F*(df)** | ***p*** | **η_p_^2^** | ***F*(df)** | ***p*** | **η_p_^2^** | ***F*(df)** | ***p*** | **η_p_^2^** |
| **Actual-shortest path length in mm (sqrt)** | **A1** | 15.23  (0-80) | 14.96 | 15.55  (0-79) | 14.61 | 15.39  (0-80) | 14.78 |  |  |  |  |  |  |  |  |  |
|  | **E1** | 10.95  (0-58) | 12.12 | 13.37  (0-83) | 14.17 | 12.16  (0-83) | 13.23 |  |  |  |  |  |  |  |  |  |
|  | **Tot** | 13.09  (0-80) | 13.78 | 14.46  (0-83) | 14.42 | 13.78  (0-83) | 14.11 |  |  |  |  |  |  |  |  |  |
|  |  |  |  |  |  |  |  | *F*(1,50) = 0.34 | .57 | .01 | ***F*(1, 50) = 8.22** | **.01** | **.14** | *F*(1,50) = 0.86 | .36 | .02 |
|  |  |  |  |  |  |  |  |  |  |  |  |  |  |  |  |  |
| **Actual-shortest path length in mm (sqrt)** | **A2** | 17.77  (0-134) | 15.98 | 17.33  (0-99) | 14.89 | 17.55  (0-134) | 15.44 |  |  |  |  |  |  |  |  |  |
|  | **E2** | 15.29  (0-79) | 12.89 | 15.34  (0-73) | 13.21 | 15.32  (0-79) | 13.04 |  |  |  |  |  |  |  |  |  |
|  | **Tot** | 16.53  (0-134) | 14.56 | 16.34  (0-99) | 14.10 | 16.43  (0-134) | 14.33 |  |  |  |  |  |  |  |  |  |
|  |  |  |  |  |  |  |  | *F*(1,50) = 0.01 | .92 | .00 | *F*(1,50) = 3.52 | .07 | .07 | *F*(1,50) = 0,04 | .84 | .00 |
|  |  |  |  |  |  |  |  |  |  |  |  |  |  |  |  |  |
|  |  |  |  |  |  |  |  |  |  |  |  |  |  |  |  |  |
|  |  |  |  |  |  |  |  |  |  |  |  |  |  |  |  |  |
|  |  |  |  |  |  |  |  |  |  |  |  |  |  |  |  |  |
|  |  |  |  |  |  |  |  |  |  |  |  |  |  |  |  |  |
|  |  | **Descriptive statistics** | | | | | | **Inferential Statistics** | | | | | | | | |
|  |  | **ASD** | | **TD** | | **Total** | | **Group** | | | **Condition** | | | **Group x Condition** | | |
| **Mea-sure** | **Con** | ***M* (range)** | ***SD*** | ***M* (range)** | ***SD*** | ***M* (range)** | ***SD*** | ***F*(df)** | ***p*** | **η_p_^2^** | ***F*(df)** | ***p*** | **η_p_^2^** | ***F*(df)** | ***p*** | **η_p_^2^** |
| **Time to target in ms (sqrt)** | **A1** | 56.69 (12-256) | 32.38 | 57.53 (13-206) | 29.98 | 57.11 (12-256) | 31.19 |  |  |  |  |  |  |  |  |  |
|  | **E1** | 49.34 (12-258) | 34.77 | 53.29 (10-198) | 32.22 | 51.31 (10-258) | 33.56 |  |  |  |  |  |  |  |  |  |
|  | **Tot** | 53.01 (12-258) | 33.78 | 55.41 (10-206) | 31.17 | 54.21 (10-258) | 32.51 |  |  |  |  |  |  |  |  |  |
|  |  |  |  |  |  |  |  | *F*(1,50) = 0.23 | .64 | .00 | ***F*(1, 50) = 5.77** | **.02** | **.10** | *F*(1,50) = 0.41 | .52 | .01 |
|  |  |  |  |  |  |  |  |  |  |  |  |  |  |  |  |  |
| **Time to target in ms (sqrt)** | **A2** | 62.20 (10-224) | 34.63 | 60.14 (12-191) | 31.61 | 61.17 (10-224) | 33.15 |  |  |  |  |  |  |  |  |  |
|  | **E2** | 58.93 (12-238) | 35.68 | 59.59 (12-253) | 33.12 | 59.26 (12-253) | 34.40 |  |  |  |  |  |  |  |  |  |
|  | **Tot** | 60.56 (10-238) | 35.17 | 59.86 (12-253) | 32.36 | 60.21 (10-253) | 33.79 |  |  |  |  |  |  |  |  |  |
|  |  |  |  |  |  |  |  | *F*(1,50) = .02 | .89 | .00 | *F*(1,50) = 0.63 | .43 | .01 | *F*(1,50) = .32 | .57 | .01 |
|  |  |  |  |  |  |  |  |  |  |  |  |  |  |  |  |  |
| **Path angle (sqrt of absolute value)** | **A1** | 5.70  (0-13) | 3.38 | 5.04  (0-13) | 3.08 | 5.37  (0-13) | 3.25 |  |  |  |  |  |  |  |  |  |
|  | **E1** | 5.50  (0-13) | 3.35 | 5.22  (0-13) | 3.18 | 5.36  (0-13) | 3.27 |  |  |  |  |  |  |  |  |  |
|  | **Tot** | 5.60  (0-13) | 3.36 | 5.13  (0-13) | 3.13 | 5.36  (0-13) | 3.26 |  |  |  |  |  |  |  |  |  |
|  |  |  |  |  |  |  |  | *F*(1,50) = 1.2 | .28 | .02 | *F*(1,50) = 0.01 | .94 | .00 | *F*(1,50) = 1.11 | .30 | .02 |
|  |  |  |  |  |  |  |  |  |  |  |  |  |  |  |  |  |
|  |  |  |  |  |  |  |  |  |  |  |  |  |  |  |  |  |
|  |  | **Descriptive statistics** | | | | | | **Inferential Statistics** | | | | | | | | |
|  |  | **ASD** | | **TD** | | **Total** | | **Group** | | | **Condition** | | | **Group x Condition** | | |
| **Mea-sure** | **Con** | ***M* (range)** | ***SD*** | ***M* (range)** | ***SD*** | ***M* (range)** | ***SD*** | ***F*(df)** | ***p*** | **η_p_^2^** | ***F*(df)** | ***p*** | **η_p_^2^** | ***F*(df)** | ***p*** | **η_p_^2^** |
| **Path angle (sqrt of absolute value)** | **A2** | 5.46  (0-13) | 3.02 | 5.12  (0-13) | 2.91 | 5.29  (0-13) | 2.97 |  |  |  |  |  |  |  |  |  |
|  | **E2** | 6.15  (0-13) | 3.06 | 5.75  (0-13) | 3.00 | 5.95  (0-13) | 3.03 |  |  |  |  |  |  |  |  |  |
|  | **Tot** | 5.81  (0-13) | 3.06 | 5.44  (0-13) | 2.97 | 5.62  (0-13) | 3.02 |  |  |  |  |  |  |  |  |  |
|  |  |  |  |  |  |  |  | *F*(1,50) = 1.07 | .31 | .02 | ***F*(1,50) = 19.13** | **.00** | **.28** | *F*(1,50) = 0.04 | .85 | .00 |

Table S3

*Descriptive and inferential statistics for control measures taken for allocentric 1, egocentric 1 (the 2 original conditions), allocentric 2 and egocentric 2 (the 2 added conditions) for individuals with autism spectrum disorder (ASD) and typical development (TD).*

|  |  | **Descriptive statistics** | | | **Inferential Statistics** | | | | | | | | |
| --- | --- | --- | --- | --- | --- | --- | --- | --- | --- | --- | --- | --- | --- |
|  |  | **ASD** | **TD** | **Total** | **Group** | | | **Condition** | | | **Group x Condition** | | |
| **Measure** | **Condition** | ***M (SD)*** | ***M (SD)*** | ***M (SD)*** | ***F*(df)** | ***p*** | **η_p_^2^** | ***F*(df)** | ***p*** | **η_p_^2^** | ***F*(df)** | ***p*** | **η_p_^2^** |
| **Out of pool times** | **Allocentric 1** | 0.00 | 0.00 | 0.00 |  |  |  |  |  |  |  |  |  |
|  | **Egocentric 1** | 0.00 | 0.00 | 0.00 |  |  |  |  |  |  |  |  |  |
|  | **Allocentric 2** | 0.00 | 0.00 | 0.00 |  |  |  |  |  |  |  |  |  |
|  | **Egocentric 2** | 0.00 | 0.00 | 0.00 |  |  |  |  |  |  |  |  |  |
|  | **Total** | 0.00 | 0.00 | 0.00 |  |  |  |  |  |  |  |  |  |
|  |  |  |  |  |  |  |  |  |  |  |  |  |  |
| **Finger lift times** | **Allocentric 1** | 0.30 (1.26) | 0.38 (1.15) | 0.34 (1.21) |  |  |  |  |  |  |  |  |  |
|  | **Egocentric 1** | 0.22 (0.79) | 0.36 (1.44) | 0.29 (1.16) |  |  |  |  |  |  |  |  |  |
|  | **Allocentric 2** | 0.38 (1.88) | 0.52 (1.70) | 0.45 (1.79) |  |  |  |  |  |  |  |  |  |
|  | **Egocentric 2** | 0.49 (2.89) | 0.47 (2.30) | 0.48 (2.61) |  |  |  |  |  |  |  |  |  |
|  | **Total** | 0.35 (1.88) | 0.43 (1.70) | 0.39 (1.79) |  |  |  |  |  |  |  |  |  |
|  |  |  |  |  | F(1,50) = 0.21 | .65 | .00 | F(3,150) = 1.17 | .32 | .02 | F(3,150) = 0.18 | .91 | .00 |
|  |  |  |  |  |  |  |  |  |  |  |  |  |  |
|  |  |  |  |  |  |  |  |  |  |  |  |  |  |
|  |  | **Descriptive statistics** | | | **Inferential Statistics** | | | | | | | | |
|  |  | **ASD** | **TD** | **Total** | **Group** | | | **Condition** | | | **Group x Condition** | | |
| **Measure** | **Condition** | ***M (SD)*** | ***M (SD)*** | ***M (SD)*** | ***F*(df)** | ***p*** | **η_p_^2^** | ***F*(df)** | ***p*** | **η_p_^2^** | ***F*(df)** | ***p*** | **η_p_^2^** |
| **Time out times** | **Allocentric 1** | 0.00 (0.05) | 0.00 (0.00) | 0.00 (0.03) |  |  |  |  |  |  |  |  |  |
|  | **Egocentric 1** | 0.01 (0.08) | 0.00 (0.00) | 0.00 (0.06) |  |  |  |  |  |  |  |  |  |
|  | **Allocentric 2** | 0.00 (0.00) | 0.00 (0.00) | 0.00 (0.00) |  |  |  |  |  |  |  |  |  |
|  | **Egocentric 2** | 0.00 (0.00) | 0.00 (0.05) | 0.00 (0.03) |  |  |  |  |  |  |  |  |  |
|  | **Total** | 0.00 (0.05) | 0.00 (0.02) | 0.00 (0.04) |  |  |  |  |  |  |  |  |  |
|  |  |  |  |  | F(1,50) = 0.84 | .37 | .02 | F(1.4,69.76) = 1.15 GG | .31 | .02 | F(1.4,69.76) = 2.11 GG | .14 | .04 |
| **First movement time in ms** | **Allocentric 1** | 2.01 (5.55) | 3.95 (9.34) | 2.98 (7.74) |  |  |  |  |  |  |  |  |  |
|  | **Egocentric 1** | 2.38 (7.03) | 3.90 (14.98) | 3.14 (11.72) |  |  |  |  |  |  |  |  |  |
|  | **Allocentric 2** | 1.96 (6.40) | 4.56 (14.89) | 3.31 (11.53) |  |  |  |  |  |  |  |  |  |
|  | **Egocentric 2** | 2.17 (7.74) | 4.19 (11.51) | 3.18 (9.85) |  |  |  |  |  |  |  |  |  |
|  | **Total** | 2.13 (6.72) | 4.17 (12.89) | 3.15 (10.33) |  |  |  |  |  |  |  |  |  |
|  |  |  |  |  | F(1,50) = 3.87 | .06 | .07 | F(3,150) = 0.22 | .88 | .00 | F(3,150) = 0.71 | .55 | .01 |
|  |  |  |  |  |  |  |  |  |  |  |  |  |  |
|  |  |  |  |  |  |  |  |  |  |  |  |  |  |
|  |  |  |  |  |  |  |  |  |  |  |  |  |  |
|  |  |  |  |  |  |  |  |  |  |  |  |  |  |
|  |  | **Descriptive statistics** | | | **Inferential Statistics** | | | | | | | | |
|  |  | **ASD** | **TD** | **Total** | **Group** | | | **Condition** | | | **Group x Condition** | | |
| **Measure** | **Condition** | ***M (SD)*** | ***M (SD)*** | ***M (SD)*** | ***F*(df)** | ***p*** | **η_p_^2^** | ***F*(df)** | ***p*** | **η_p_^2^** | ***F*(df)** | ***p*** | **η_p_^2^** |
| **Time for distracter task in ms** | **Allocentric 1** | 14639.5 (7836.9) | 13248.3 (3322.19) | 13943.9 (6055.39) |  |  |  |  |  |  |  |  |  |
|  | **Egocentric 1** | 10142.1 (5554.5) | 9497.31 (3346.64) | 9819.72 (4594.02) |  |  |  |  |  |  |  |  |  |
|  | **Allocentric 2** | 9916.53 (4574.2) | 9227.05 (2744.02) | 9571.79 (3785.28) |  |  |  |  |  |  |  |  |  |
|  | **Egocentric 2** | 13683.4 (5031.57) | 13257.5 (3152.25) | 13470.4 (4201.3) |  |  |  |  |  |  |  |  |  |
|  | **Total** | 12095.4 (6241.53) | 11307.5 (3701.85) | 11701.5 (5145.63) |  |  |  |  |  |  |  |  |  |
|  |  |  |  |  | F(1,50) = 1.11 | .30 | .02 | **F(2.36,117.75) = 152.92 GG** | **.00** | **.75** | F(2.36,117.75) = 1.24 | .30 | .02 |

**Place learning**

**ASD**

**Trial 1 Trial 16**

**
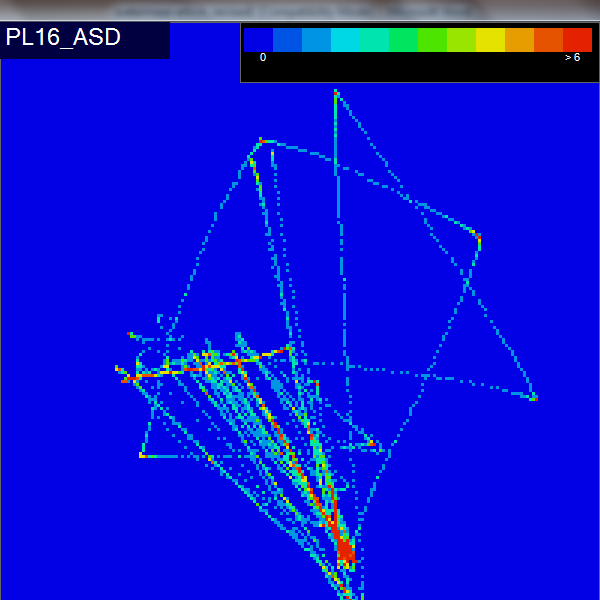
**

**
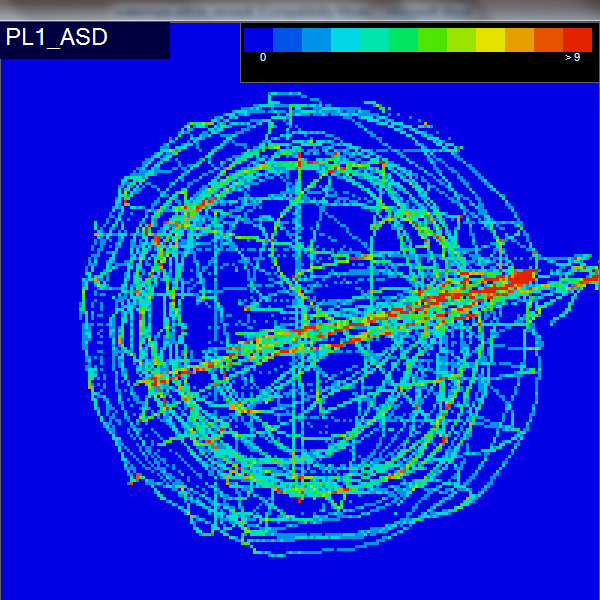
**

**TD**

**Trial 1 Trial 16**


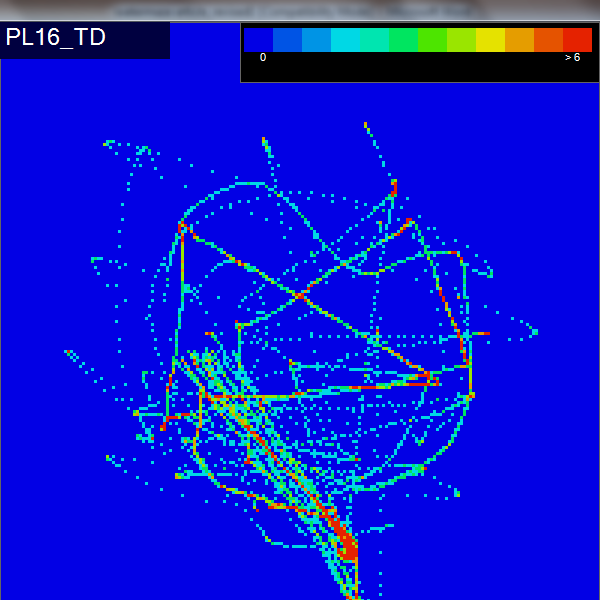


**
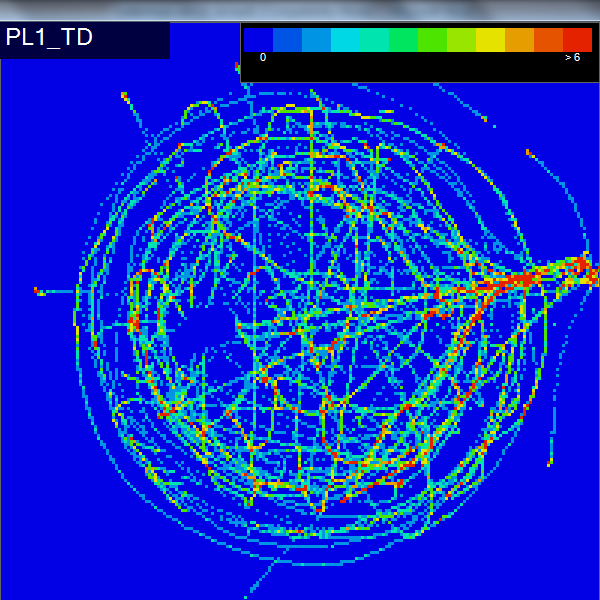
**

**Allocentric 1**

**ASD**

**Trial 1 Trial 16**


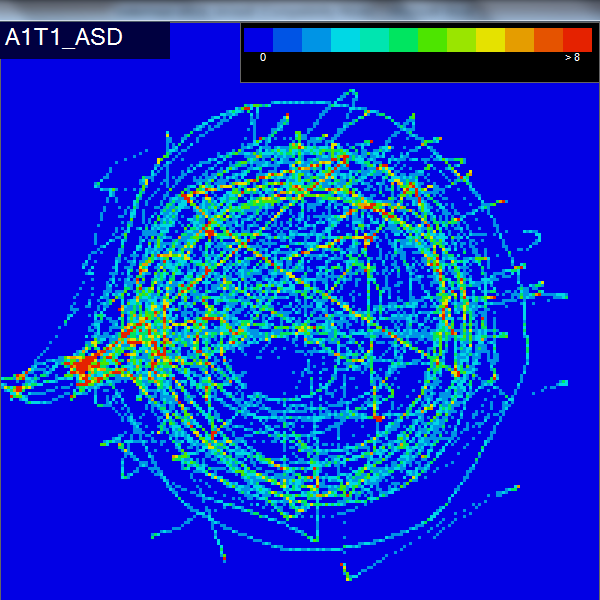


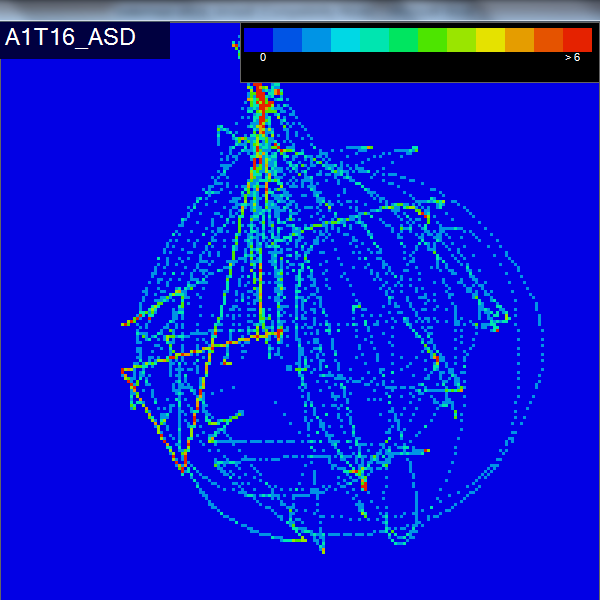


**TD**

**Trial 1 Trial 16**


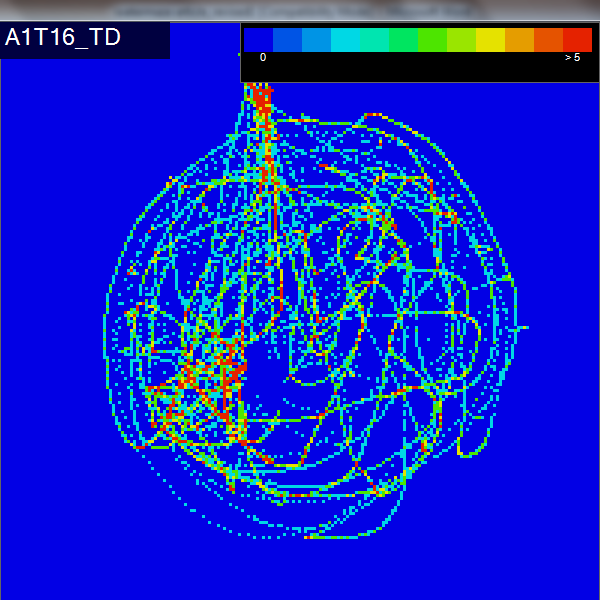


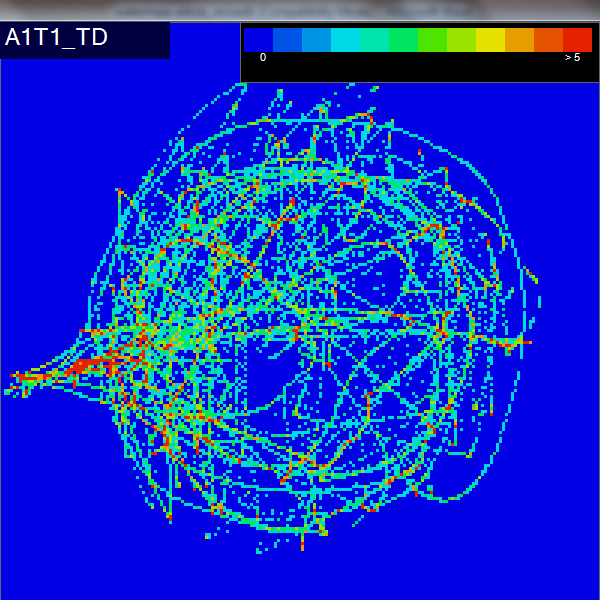


**Egocentric 1**

**ASD**

**Trial 1 Trial 16**


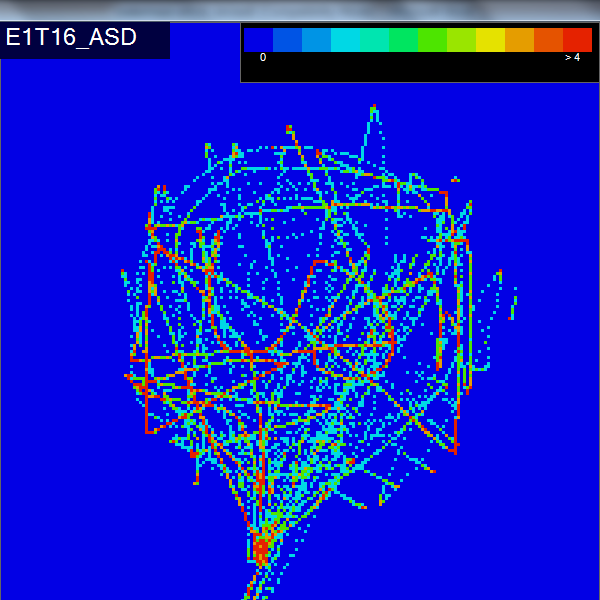


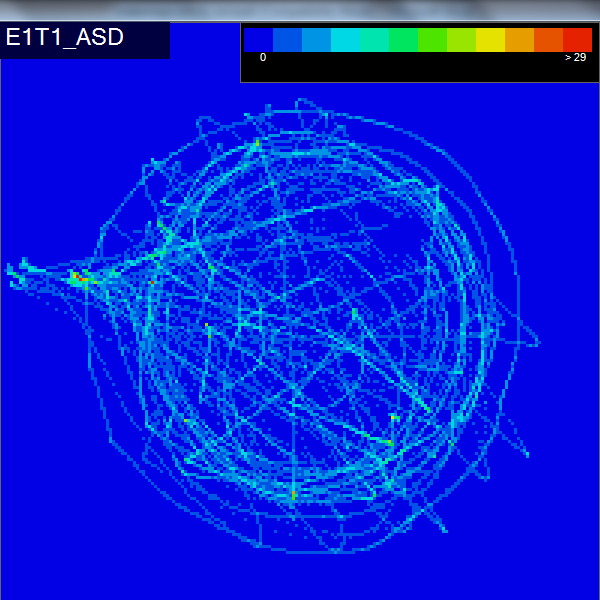


**TD**

**Trial 1 Trial 16**


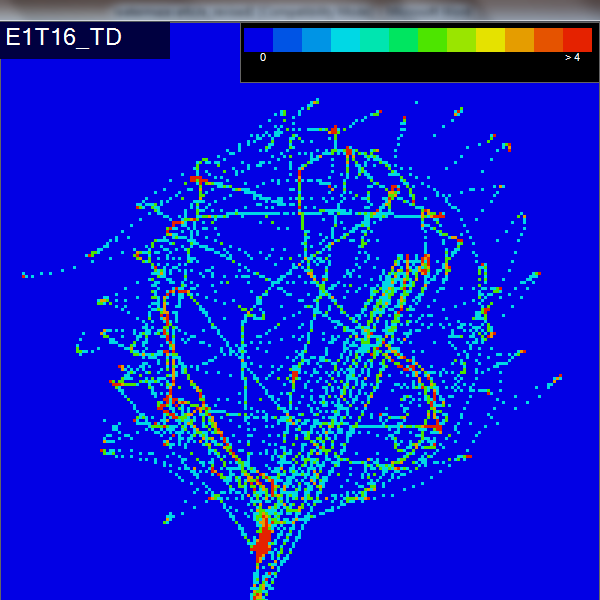


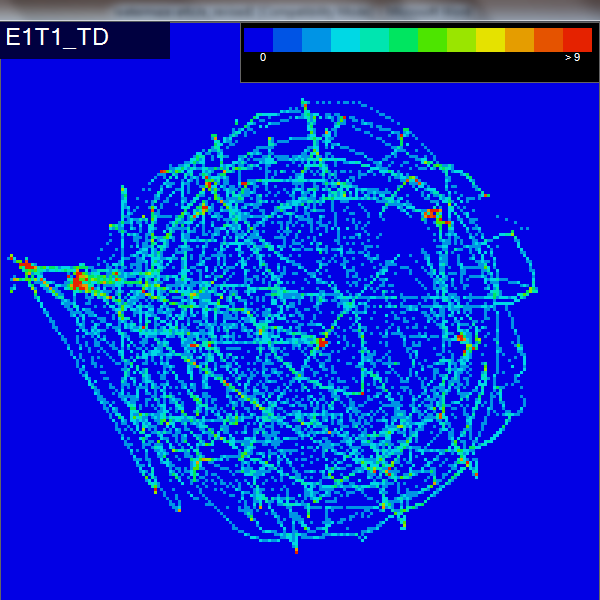


**Allocentric 2**

**ASD**

**Trial 1 Trial 16**


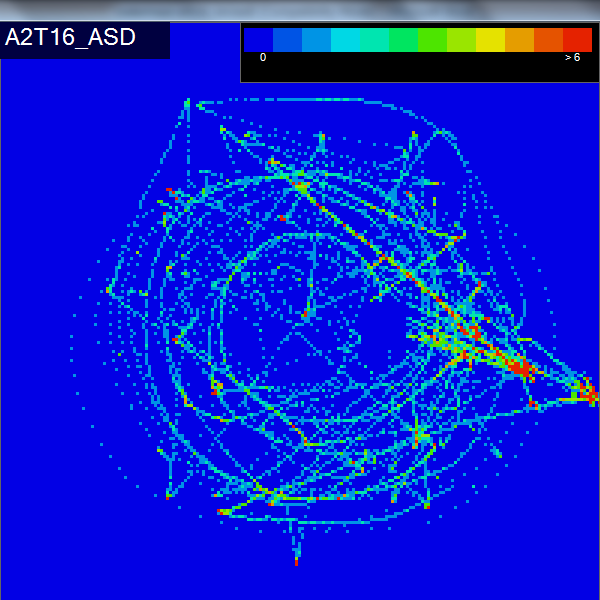


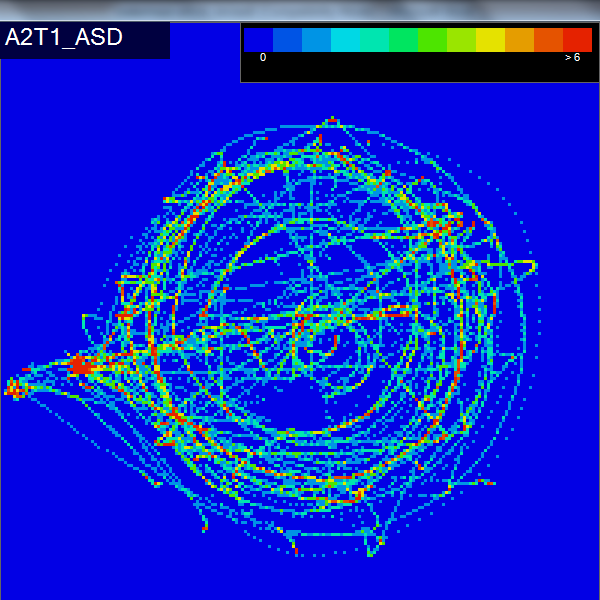


**TD**

**Trial 1 Trial 16**


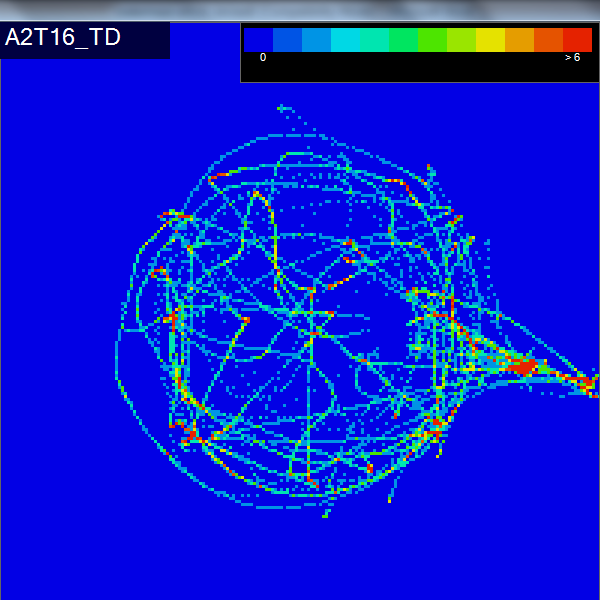


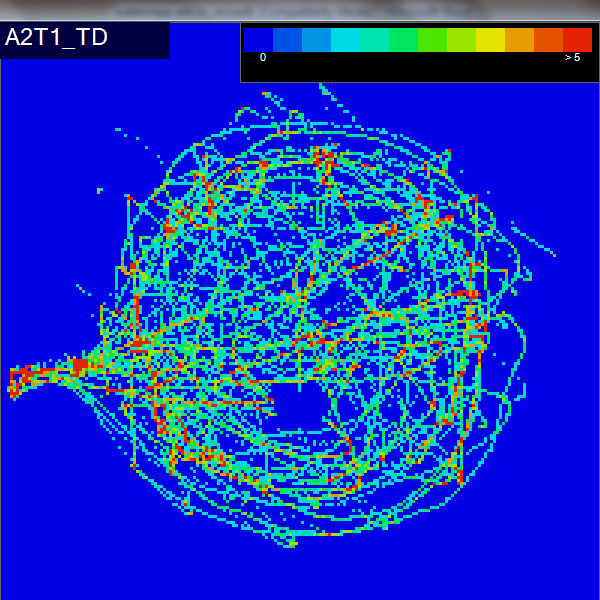


**Egocentric 2**

**ASD**

**Trial 1 Trial 16**


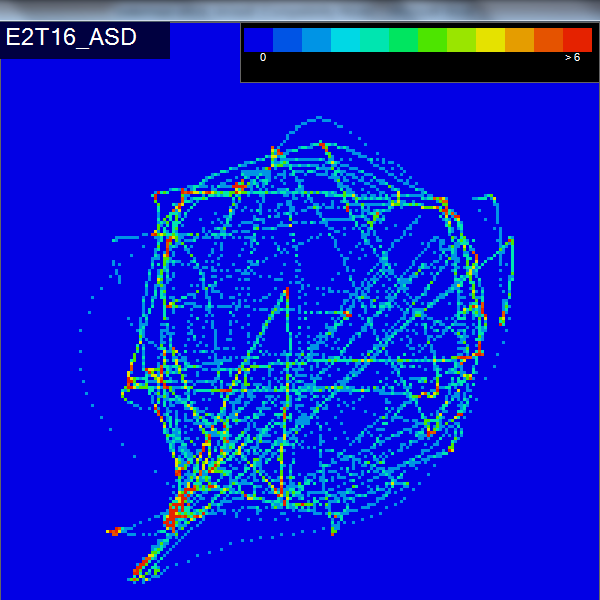


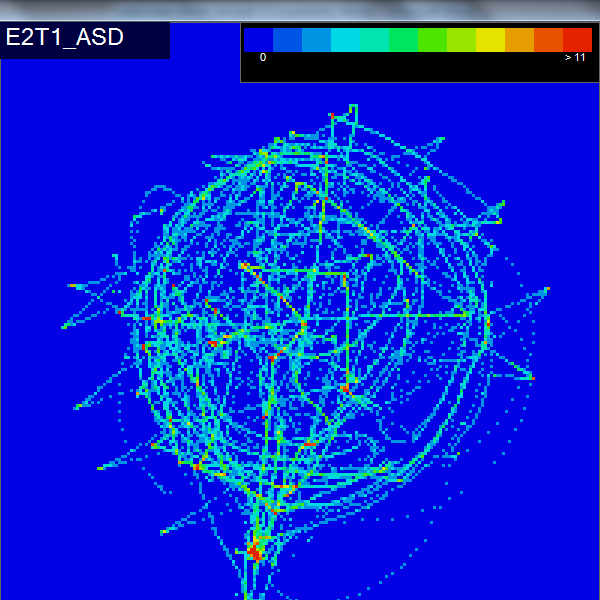


**TD**

**Trial 1 Trial 16**


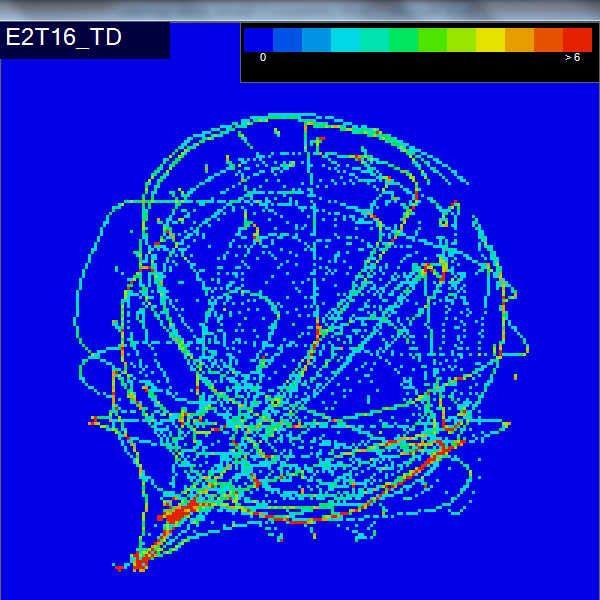


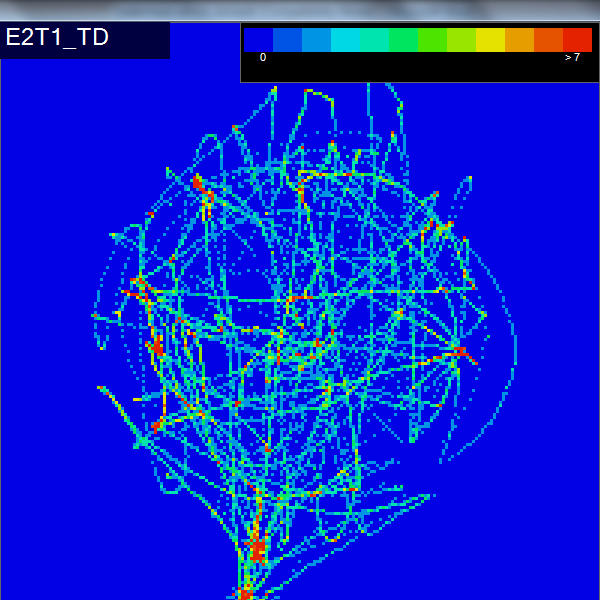


*Figure S1.* Heat maps presenting the paths taken to find the platform for place learning, allocentric 1, egocentric 1, allocentric 2 and egocentric 2 for individuals with autism spectrum disorder (ASD) and typical development (TD) comparing performance on the first and the last trials of every condition.
